# Targeted proteomic analysis of 3R and 4R tau in marmoset and human brain tissue

**DOI:** 10.1002/alz.092505

**Published:** 2025-01-03

**Authors:** Sarah M Shapley, Hasi Huhe, Duc M Duong, Fang Wu, Gregory W Carter, Afonso C Silva, Stacey J Sukoff Rizzo, Nicholas T Seyfried

**Affiliations:** ^1^ Emory University School of Medicine, Atlanta, GA USA; ^2^ University of Pittsburgh School of Medicine, Pittsburgh, PA USA; ^3^ The Jackson Laboratory, Bar Harbor, ME USA

## Abstract

**Background:**

The microtubule‐associated Tau gene (*MAPT*) undergoes alternative splicing to produce isoforms with varying combinations of microtubule‐binding region (MTBR) repeats (3R, 4R). The MTBR is the predominant region that forms paired helical filaments and neurofibrillary tangles fibrils in disease. Alzheimer’s disease (AD) is a mixed Tauopathy containing both 3R and 4R isoforms. Previously, marmosets have been shown to spontaneously develop the key pathological hallmarks of AD during advanced age, positioning them as a model system to overcome the rodent‐to‐human translational gap for AD. Yet, Tau isoform expression has been understudied in marmosets with conflicting reports of isoform expression. Recently, our lab found 3R and 4R Tau to be expressed in adult marmoset brains. Additionally, AD pathology related phospho‐epitopes of soluble (pT181, pT217, pT231) and insoluble (pS396/404 and pS202/205) Tau were conserved. The present study sought to comprehensively characterize Tau isoforms in marmosets and corroborate antibody‐based results with a targeted proteomics assay.

**Method:**

Marmoset (n = 7), human AD (n = 4), human non‐demented control (n = 4), and mouse (n = 1) brain lysates were analyzed via targeted mass spectrometry. The detection of the 3R Tau isoform unique peptide, KVQIVY, was obtained through the use of the LysargiNase enzyme that cleaves N‐terminal to K/R residues. Recombinant 3R and 4R Tau (rTau) proteins were used to generate peptide spectral libraries and served as technical controls.

**Result:**

Tau peptides were identified via retention time and co‐elution. The marmoset product ions mirrored the human peaks and highly corresponded to the 3R and 4R peptide library spectra, providing mass spectrometry evidence of both isoform expressions in marmoset brains. 3R Tau was enriched in the fetal marmoset. In conjunction, the 4R rTau isoform and mouse samples did not match the 3R recombinant library spectrum.

**Conclusion:**

Overall, we found marmosets to be a promising translational model of AD that overcomes the limitations of mice concerning the expression of both 3R and 4R Tau isoforms. These data extend our previous findings from western blots in a cohort of unrelated marmosets. Additionally, proteotyping of other Tauopathies may lend insight for novel biomarkers and personalized therapeutics.

